# What Is the Evidence for Using Anti-Epileptic Drugs to Treat Agitation and Irritability in Huntington's Disease?

**DOI:** 10.1192/bjo.2022.505

**Published:** 2022-06-20

**Authors:** Kok Keong Leong, Mark Paramlall

**Affiliations:** Cygnet Brunel, Bristol, United Kingdom

## Abstract

**Aims:**

Background: Huntington's disease (HD), due to the pathological expansion of CAG trinucleotide repeats within the Huntington's gene on chromosome 4p (1), is an autosomal dominant progressive neurodegenerative disease with motor, cognitive and neuropsychiatric symptoms that includes irritability and agitation in an estimated 38–73% of HD patients (2) which is characterized by impatience and a tendency to become angry in response to minimal provocation. Expert consensus recommends implementation of environmental and behavioural strategies then commencing treatment with SSRI's, Neuroleptics or Anti-Epileptic Drugs (AED) (3). No previous papers, to our knowledge, have examined the newest antiepileptic agents or have identified the most efficacious antiepileptics for this use. Aim: We present a review of the literature describing antiepileptic treatments for agitation and irritability in HD focusing on identification of the most efficacious antiepileptics and the role of newer antiepileptic agents for this use.

**Methods:**

A search in the main database sources (EMBASE, MEDLINE, Allied and Complementary medicine) was performed in order to obtain a comprehensive evaluation of available antiepileptic psychopharmacological treatments in HD for agitation and irritability.

**Results:**

Antiepileptic (AED) agents described in consensus statements and case studies have included sodium valproate, carbamazepine and lamotrigine, which work by inhibition of voltage gated Na and Ca channels, and are often combined with antipsychotic agents for improvement of pathological mood swings and irritability. However, none of the papers identified were Level III or better.

**Conclusion:**

No specific mood stabilizing antiepileptic psychopharmacological treatment of the Psychiatric symptoms of irritability and agitation in HD was identified. Overall, the use of AED have weak evidence base with no quantifiable outcome measures, such as such as the Disruptive or Aggressive Behavior behavioural subscale of the Unified Huntington's Disease Rating Scale or the Neuropsychiatric Inventory, indicating improvement of symptoms identified. Surveys and expert opinions were based on their personal knowledge of the HD populations and the selection of the experts surveyed was not systematic which could influence the practice pattern results. The review indicates a pressing need for treatment studies to determine which psychopharmacological and behavioral treatments are most efficacious.

